# Value of corneal epithelial and Bowman’s layer vertical thickness profiles generated by UHR-OCT for sub-clinical keratoconus diagnosis

**DOI:** 10.1038/srep31550

**Published:** 2016-08-11

**Authors:** Zhe Xu, Jun Jiang, Chun Yang, Shenghai Huang, Mei Peng, Weibo Li, Lele Cui, Jianhua Wang, Fan Lu, Meixiao Shen

**Affiliations:** 1 School of Ophthalmology and Optometry, Wenzhou Medical University, Wenzhou, Zhejiang, China; 2 Department of Ophthalmology, Bascom Palmer Eye Institute, University of Miami, Miami, FL, USA

## Abstract

Ultra-high resolution optical coherence tomography (UHR-OCT) can image the corneal epithelium and Bowman’s layer and measurement the thicknesses. The purpose of this study was to validate the diagnostic power of vertical thickness profiles of the corneal epithelium and Bowman’s layer imaged by UHR-OCT in the diagnosis of sub-clinical keratoconus (KC). Each eye of 37 KC patients, asymptomatic fellow eyes of 32 KC patients, and each eye of 81 normal subjects were enrolled. Vertical thickness profiles of the corneal epithelium and Bowman’s layer were measured by UHR-OCT. Diagnostic indices were calculated from vertical thickness profiles of each layer and output values of discriminant functions based on individual indices. Receiver operating characteristic curves were determined, and the accuracy of the diagnostic indices were assessed as the area under the curves (AUC). Among all of the individual indices, the maximum ectasia index for epithelium had the highest ability to discriminate sub-clinical KC from normal corneas (AUC = 0.939). The discriminant function containing maximum ectasia indices of epithelium and Bowman’s layer further increased the AUC value (AUC = 0.970) for sub-clinical KC diagnosis. UHR-OCT-derived thickness indices from the entire vertical thickness profiles of the corneal epithelium and Bowman’s layer can provide valuable diagnostic references to detect sub-clinical KC.

Keratoconus (KC) is usually a bilateral and progressive corneal disease characterized by keratectasia and by thinning and increased curvature of the cornea^1^. The distorted corneal structure reduces the optical quality of the eye, making it difficult to correct with spectacles or contact lenses^2^. Because unidentified sub-clinical KC is the main cause of iatrogenic keratectasia after laser-assisted *in-situ* keratomileusis (LASIK)^3^,^4^,^5^, early diagnosis of sub-clinical KC is important in patients seeking corneal refractive surgery.

In KC, the epithelium thins over the cone area, and in advanced KC, it can lead to a breakdown of the epithelium^6^,^7^. Epithelial thinning and thickness irregularity have been demonstrated *in vitro* by histopathologic analysis and by light microscope observation^8^,^9^. In addition to the epithelial changes, disruption of Bowman’s layer, including splitting, occurs in the cone region^9^,^10^,^11^. These changes can result in a scar at the apex of the cornea during progression of the disease^9^,^12^.

*In vivo* imaging modalities, such as confocal microscopy, ultrasound, and optical coherence tomography (OCT), provide insight into the corneal sublayer abnormalities occurring in KC patients, thereby improving the evaluation and diagnosis of the disease^13^,^14^,^15^,^16^. Bowman’s layer breaks and discontinuities in manifest KC can be imaged by confocal microscopy and ultrasound, both of which are minimally invasive but have limited axial resolution^8^,^15^,^17^. In contrast, OCT is noninvasive and has high resolution based on the principles of low-coherence interferometry^18^. The high axial resolution of Fourier-domain OCT provides a distinct image showing the epithelium, Bowman’s layer, stroma, Descemet’s membrane, and endothelium, permitting accurate measurements of axial thickness^7^,^14^,^16^. Recently, Li *et al.*, using a commercially available high resolution OCT instrument, reported thinning of the central corneal epithelium in manifest KC^16^. Abou Shousha *et al.* used ultra-high resolution OCT (UHR-OCT) to identify localized thinning of Bowman’s layer as a diagnostic feature of KC^14^. Both of these studies used diagnostic indices to specifically quantify the irregular alterations of the topographic thickness of the epithelium and Bowman’s layer. With OCT, there is high sensitivity in discriminating manifest KC from normal corneas^14^,^16^,^19^,^20^,^21^. However, to the best of our knowledge, the characteristic thickness changes and the diagnostic values of the corneal sub-layers in the sub-clinical stage of KC remain unreported^22^. The purpose of this study was to measure entire vertical thickness profiles of the epithelium and Bowman’s layer in sub-clinical KC, KC, and normal corneas using UHR-OCT. Based on the characteristic changes in thickness of the epithelium, Bowman’s layer, and stroma, we sought to develop indices that could identify sub-clinical KC and discriminate it from normal eyes.

## Results

### Demographics

Data were analyzed for one eye each of 37 KC patients (25 men and 12 women, average age ± standard deviation 23.9 ± 5.6 years), the asymptomatic fellow eye of 32 KC patients (20 men and 12 women, age 20.5 ± 5.5 years), and one eye each of 81 normal subjects (46 men and 35 women, 25.4 ± 2.6 years). Table 1 summarizes the different characteristics of the three groups. The maximum keratometry (Max-K), minimum keratometry (Min-K), average keratometry (Avg-K), and astigmatic keratometry (Ast-K) in the KC group were significantly higher than in the other two groups (analysis of variance [ANOVA], P < 0.05, Table 1).

### Intergroup Differences: Thickness Profiles of the Corneal Epithelium, Bowman’s Layer, and Stroma

**Figure 1:**
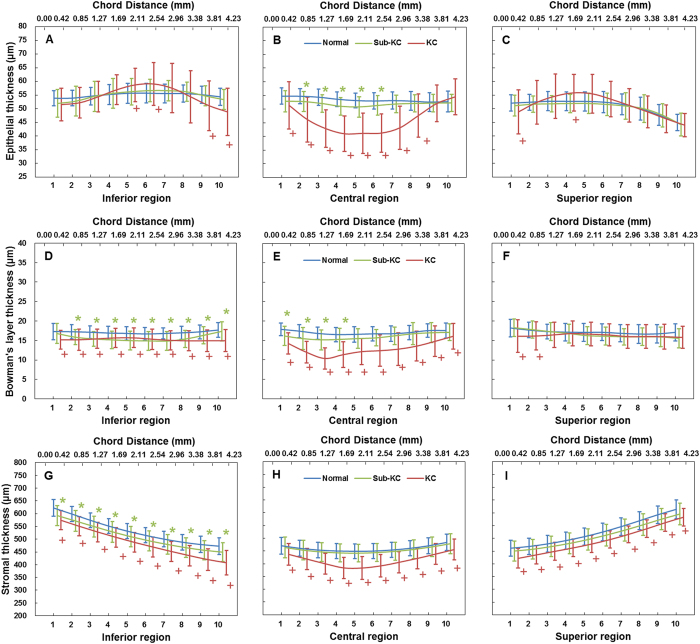
Averaged vertical thickness profiles of the entire epithelium, Bowman’s layer, and stroma for normal, sub-clinical keratoconus, and keratoconus groups. (**A**) Inferior epithelial thickness profiles. (**B**) Central epithelial thickness profiles. (**C**) Superior epithelial thickness profiles. (**D**) Inferior Bowman’s layer thickness profiles. (**E**) Central Bowman’s layer thickness profiles. (**F**) Superior Bowman’s layer thickness profiles. (**G**) Inferior stromal thickness profiles. (**H**) Central stromal thickness profiles. (**I**) Superior stromal thickness profiles. The thickness result of each scan was determined from 1,000 A-scans, equivalent to a chord distance of 4.23 mm (upper scale), and was divided into 10 zones (lower scale). Zones 1 to 10 represent the direction from inferior to superior. Bars, standard deviation. *significant differences between normal and sub-clinical KC groups (P < 0.05). +significant differences between normal and KC groups (P < 0.05).

Compared to normal eyes, there was no significant thinning of the inferior epithelium in sub-clinical KC eyes (Fig. 1A). However, there was significant thinning of the corneal epithelium in the central region for both the KC (ANOVA, P = 0.01, Table 2, Fig. 1B) and the sub-clinical KC (ANOVA, P < 0.05, Table 2, Fig. 1B) groups. Among all the zones with significant thickness differences compared with the normal group, the thinnest epithelium was 50.81 ± 3.73 μm located in zone 5 of the central region (central 1.69 to 2.11 mm) for sub-clinical KC (Table 2, Fig. 1B) and 40.97 ± 6.51 μm located in zone 4 of the central region (central 1.27 to 1.69 mm) for KC patients (Table 2, Fig. 1B).

Both sub-clinical KC and KC eyes had thinner Bowman’s layers in the inferior region compared to the normal control eyes (ANOVA, P < 0.05 and P < 0.01 for sub-clinical KC and KC, Table 2, Fig. 1D). Bowman’s layer in the KC group was significantly thinner in the central region (ANOVA, P < 0.01, Table 2, Fig. 1E). Among all the zones with significant thickness differences with the normal group, the thinnest Bowman’s layer was 14.85 ± 2.42 μm in zone 7 of the inferior region (inferior 2.54 to 2.96 mm) for sub-clinical KC eyes (Table 2, Fig. 1D) and 10.37 ± 2.69 μm in zone 3 of the central region (central 0.85 to 1.27 mm) for KC eyes (Table 2, Fig. 1E).

The stromal thickness of the sub-clinical KC eyes was thinner in the inferior region compared with the normal eyes (ANOVA, P < 0.05, Table 2, Fig. 1G). The KC eyes had a thinner stromal thickness for the entire profile (ANOVA, P < 0.01, Table 2, Fig. 1G,H, and I). In the KC group, among all the zones with significant differences from the normal group, the thinnest stromal thickness was 383.82 ± 41.84 μm in zone 4 of the central region (central 1.27 to 1.69 mm) (Table 2, Fig. 1H).

### ROC Analysis

The detailed definitions of the diagnostic thickness ectasia indices for the epithelium, Bowman’s layer, and stroma (EEI, BEI, and SEI respectively), the maximum ectasia indices for each layer (EEI-MAX, BEI-MAX, and SEI-MAX), the profile variations within the layer deviations (EPV, BPV, and SPV), and the standard deviations from normal patterns (EPSD, BPSD, and SPSD) are shown in Table 3.

**Figure 2:**
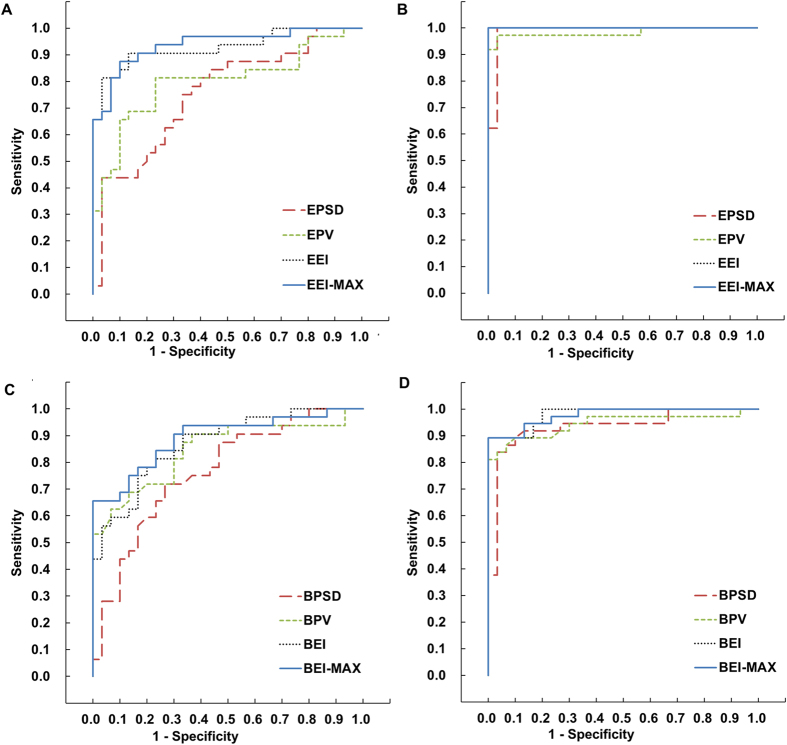
Receiver operating characteristic (ROC) curve of the epithelium diagnostic indices for normal, sub-clinical keratoconus, and keratoconus groups. (**A**) ROC curve of epithelial diagnosis indices for sub-clinical KC group versus normal group. (**B**) ROC curve of epithelial diagnosis indices for KC group versus normal group. (**C**) ROC curve of Bowman’s layer diagnosis indices for sub-clinical KC group versus normal group. (**D**) ROC curve of Bowman’s layer diagnosis indices for keratoconus group versus normal group. EEI, epithelium ectasia index; EEI-MAX, maximum epithelium ectasia index; EPSD, epithelium profile standard deviation; EPV, epithelium profile variation; BEI, Bowman’s layer ectasia index, BEI-MAX, maximum Bowman’s layer ectasia index; BPSD, Bowman’s layer profile standard deviation; BPV, Bowman’s layer profile variation.

The ROC curve for each diagnostic index from the vertical thickness profiles of the corneal epithelium illustrated the discriminative ability among eyes with KC, sub-clinical KC, and normal corneas (Fig. 2A,B). All epithelial indices discriminated between sub-clinical KC and normal eyes with an area under the ROC curve (AUCs) higher than 0.75 (Table 4). Among the individual indices, EEI-MAX was the highest index for sub-clinical KC diagnosis (AUC = 0.939; sensitivity = 88%; specificity = 90%, Table 4). All of the indices of Bowman’s layer thickness had a high ability to discriminate between KC corneas and normal control corneas (Fig. 2C,D). Only the BEI and BEI-MAX obtained AUCs higher than 0.85 (Table 4). The individual indices of stromal thickness differentiated only between the KC group and normal controls (ANOVA, P < 0.05, Table 4). There were no significant differences between the stromal indices of sub-clinical KC and normal eyes (Table 4).

Based on the results of linear stepwise discriminant analysis, the EEI-MAX and BEI-MAX were included to build the discriminant function as follows:


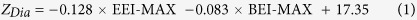


where *Z*_*Dia*_ was the discriminant function of linear stepwise discriminant analysis. The output value of discriminant function for sub-clinical KC was 1.00 ± 1.15, and for KC eyes it was 4.67 ± 2.16. Both values were significantly lower than the value for normal control eyes, −1.23 ± 0.63 (ANOVA, P < 0.05). The output value of the discriminant function showed a greater ability to discriminate sub-clinical KC eyes from normal eyes compared to each individual index (AUC = 0.970; sensitivity = 91%; specificity = 93%).

## Discussion

Previous studies indicated that in some degenerative corneal diseases such as KC, the altered epithelial thickness could compensate for the corneal surface irregularity^16^,^17^. While the epithelial thinning is evident in manifest KC^8^,^15^,^16^,^19^,^20^,^21^, changes may occur before the irregular surface can be detected by corneal topography. Yadav *et al.* used a custom-developed UHR-OCT instrument with an axial resolution of 1.1 μm in corneal tissue to image the central 4 mm of the cornea and found evidence of vertical central epithelial thinning in KC eyes^7^. Li *et al.* used a Fourier-domain OCT system, the RTVue OCT (Optovue, Inc., Fremont, CA, USA) with an axial resolution of 5 μm in corneal tissue, to map the central 6-mm corneal epithelial thickness^16^. They also found irregular epithelial thinning in the central cornea. Both studies reported the epithelial changes in only the central 4–5 mm diameter of the cornea^16^. Reinstein *et al.*, using very high-frequency ultrasound to measure the thickness of the corneal center and periphery, observed a “doughnut pattern” of epithelial thickness profile in KC patients^15^. This pattern is due to the thinning of the epithelium in the center and thickening in the periphery. This particular pattern was also detected by UHR-OCT in our current study.

We also found central epithelial thinning in the vertical meridian of the sub-clinical KC group. The changes in epithelial thickness, as reflected in the EPSD, EPV, EEI, and EEI-MAX, showed that the changes in profile developed in the sub-clinical stage of KC. The alterations may occur even in the eyes that do not show severe abnormalities with corneal topography examination. Temstet *et al.*, using the RTVue OCT system, reported that the zone of minimum epithelial thickness was located inferiorly and corresponded with the thinnest corneal zone in form fruste keratoconus eyes^23^. They also concluded that the epithelium was thinnest in the central corneal zone and that location was useful for detection of form fruste keratoconus, which is consistent with our current study. Similar to previous reports^9^,^13^,^17^,^24^, epithelial thinning occurred in the very early stages of KC, suggesting that it may play an important role in compensating for the irregular stroma and help to maintain the regularity of anterior corneal surface during the disease process.

Using UHR-OCT, we observed central and inferior thinning of Bowman’s layer in KC eyes, which is consistent with previous *in vivo* findings^7^ and with *in vitro* histopathologic studies^6^,^7^. Using a similar UHR-OCT system, Abou Shousha *et al.* reported that the average Bowman’s layer profile thickness of normal eyes was 15 ± 1 μm^14^. That value is thinner than what we found, but the difference could be attributed to differences in the study population. Interestingly, the same group reported a thickness of 17.7 ± 1.6 μm for the central Bowman’s layer thickness^7^,^25^. In addition, Yadav *et al.* reported that the central Bowman’s layer thickness was 16.7 ± 2.6 μm in normal eyes^7^,^25^. Both of these results are similar to ours^7^,^25^.

In the present study, we further found that the sub-clinical KC group had decreased values for BEI and BEI-MAX, indices that represent surface shape changes due to inferior localized thinning of Bowman’s layer. The sub-clinical KC group also had increased values for BPV and BPSD, indices that represent Bowman’s layer irregularity. We hypothesize that the presence of abnormal Bowman’s layer thickness in sub-clinical KC might be caused by alterations in the lamellar structure of Bowman’s layer collagen fibers. Such changes in collagen fibers have been reported in previous studies using anterior segment polarization-sensitive OCT^26^ and X-ray scattering methods^27^,^28^. Modifications of the KC cornea would influence the lamellar structure of collagen fibers in Bowman’s layer, which in turn could eventually alter the corneal microstructures and mechanical stability^29^. Our findings on the sub-clinical KC patients support the idea that the characteristics of Bowman’s layer thickness may be an early marker for KC diagnosis.

Several previous studies evaluated the diagnostic power of indices constructed from thickness maps of the corneal epithelium and Bowman’s layer for KC and normal eyes^14^,^16^,^30^. Li *et al.* demonstrated that the root-mean-square pattern deviation of central 5-mm epithelium thickness maps provided good diagnostic power^16^. Abou Shousha *et al.* reported that the BEI-MAX index has a very high ability to discriminate KC from normal corneas^14^. With a similar UHR-OCT system, they used the image range of 3-mm diameter to reconstruct corneal profiles. The diameter used in our study was 4.23 mm, which was wider and contained overlapping images of the peripheral and central regions. The image quality enabled the recognition of corneal structures within the 4.23-mm diameter. The wider area provides more information for the diagnostic indices of sub-clinical KC detection. In the current study, we demonstrated that thickness indices constructed from the thickness profiles of the epithelium and Bowman’s layer can discriminate KC and sub-clinical KC from normal eyes. The diagnostic ability of the indices to discriminate between the manifest KC and normal eyes was consistent with results in previous studies^14^,^16^.

As expected, when applied to the sub-clinical KC group, these indices were less effective in discriminating their corneas from normal ones, possibly due to the minimal changes in the affected group. Another potential reason for the lower ability to discriminate between the sub-clinical KC eyes and normal ones is the small sample size of sub-clinical KC eyes. The small sample size might increase variability of the standard deviations and include more biases. Further, device calibration might also help to improve the accuracy of thickness measurements and index diagnostic values. Compared with the air-epithelium and epithelium-Bowman’s layer interfaces, the identification of the Bowman’s layer-stroma interface was more difficult using automated detection. Considering that the measurement repeatability for the epithelium and Bowman’s layer were similar, the thickness values and indices of the epithelium would be more precise and accurate. All of these factors might account for the lower discriminative ability of the indices constructed from the Bowman’s layer thickness profile for sub-clinical KC eyes, compared to the indices constructed from the epithelium thickness profile.

Our results showed that the discriminant function containing EEI-MAX and BEI-MAX yielded the highest AUC for discrimination of sub-clinical KC from normal corneas. This suggests that the combination of these two indices improved the detection sensitivity and specificity for sub-clinical KC. Because the commercially available OCT instruments such as RTVue OCT are able to resolve the interfaces of the epithelium and Bowman’s layer, it is likely that other commercially available OCT instruments could also image these tissues with sufficient resolution to derive the indices reported here.

Several indices have been reported to discriminate sub-clinical KC eyes from normal ones, such as KISA%, the Zernike decomposition method of corneal interfaces, corneal pachymetric distribution (Ambrósio Relational Thinnest [ART]), and the corneal elevation components (Belin/Ambrósio Enhanced Ectasia Display [BAD])^31^,^32^,^33^,^34^,^35^,^36^. Interestingly, a global consensus was reached among experts from four international corneal societies^36^. The presence of abnormal posterior ectasia, abnormal corneal thickness distribution, and clinical noninflammatory corneal thinning are mandatory elements to diagnose KC. The definition of corneal ectasia procession includes at least two of the following parameters: steepening of the anterior corneal surface, steepening of the posterior corneal surface, and progressive thinning and/or an increase in the rate of corneal thickness change from the periphery to the thinnest point. True unilateral KC does not exist. Therefore, prompt treatment in time can save vision from further damage. However, early intervention imposes greater diagnostic challenges^36^. One of these challenges is to guarantee the identification of the earliest KC with absolute accuracy. In the future, elaborate combined indices of multiple corneal features of the entire cornea may be needed to further investigate and optimize the screening protocol for sub-clinical KC.

Regarding the inclusion of sub-clinical KC in the study, we used the asymptomatic fellow eye of unilateral KC patients as one of the criteria. None of the eyes in the sub-clinical KC group showed any clinical signs of KC at slit-lamp biomicroscopy, retinoscopy, or ophthalmoscopy. Also, there were no significant differences in the keratometry results for the sub-clinical and normal groups. In previous studies, Bühren *et al.*^37^. and De Sanctis *et al.*^38^. used similar inclusion criteria for the sub-clinical KC group. In addition, based on corneal interface morphology and pachymetry, they identified various diagnostic indices for sub-clinical KC discrimination. In the present study, the UHR-OCT-derived profile indices of the entire corneal epithelium and Bowman’s layer vertical thicknesses also provided valuable diagnostic references for detecting sub-clinical KC. Thus, selection of the sub-clinical KC group validated the diagnostic power of the vertical thickness profiles of the corneal epithelium and Bowman’s layer as imaged by UHR-OCT.

This is our first attempt to investigate the characteristic patterns of epithelial and Bowman’s layer thickness changes in sub-clinical KC eyes, and the following are some limitations to our approach: (1) We only evaluated the thickness changes along the vertical scan. The thickness changes related to KC may occur in other regions around the cornea as well, so using only the vertical line scan protocol may limit our understanding of these changes. (2) The manual outlining of Bowman’s layer may have induced some variation in the measured thickness. (3) The group information was not disclosed in the OCT image names; nevertheless, the grader may not have been totally blinded to the group information during image processing because it could have been guessed based upon the corneal distortion apparent in the OCT images. (4) The sample size of sub-clinical KC eyes was small and the study design was cross-sectional. (5) The normal group only included the subjects with myopia <−6.00 diopter (D) and astigmatism <−2.00 D, which might have reduced the deviation of the normal range. (6) Corneal warpage appears to be gradually reversible after discontinuation of contact lens wear. Tsai *et al.* reported that the discontinuation of rigid gas permeable (RGP) lens wear for six weeks may be necessary to ensure refractive stability before the initial examination for surgical correction of refractive error^39^. Hashemi *et al.* reported that a two-week soft contact lens-free period seemed to be adequate for the cornea to stabilize^40^. Copeland *et al.* also concluded that the discontinuation of soft lens wear for one week and of RGP lens wear for three weeks were needed prior to refractive surgery screening^41^. Thus, there appears to be no consensus for the duration of contact lens discontinuation for corneal stabilization. In the present study, only the normal group had a history of contact lens wear. One subject stopped wearing RGP lenses for 22 days, and the other two subjects stopped wearing soft contact lenses for 9 and 10 days respectively before the examinations. Although longer periods of contact lens discontinuation would be better, the small portion of contact lens users in our study is unlikely to have significantly impacted the conclusions. (7) The UHR-OCT system captured the entire corneal profile of the epithelium and Bowman’s layer in a single shot. Overlapping image areas caused by registration existed during imaging processing. With new developments of OCT imaging technology, further studies employing longitudinal observations based on three-dimensional volume scans covering the entire cornea with larger sample sizes will be more convincing.

In summary, we demonstrated the diagnostic value for sub-clinical KC detection by using UHR-OCT to generate vertical thickness profiles of the corneal epithelium and Bowman’s layer. Sub-clinical KC was characterized by localized central epithelial and inferior Bowman’s layer thinning. The diagnostic power of indices constructed from the thickness profiles was evident in the discrimination of sub-clinical KC from normal subjects. UHR-OCT-derived thickness indices of entire vertical thickness profiles of the corneal epithelium and Bowman’s layer will be a valuable diagnostic reference for detecting sub-clinical KC.

## Methods

### Subjects

The study was approved by the Office of Research Ethics, Wenzhou Medical University. Written informed consent was obtained from each subject after the study purpose and characteristics had been explained. The tenets of the Declaration of Helsinki were followed for all study procedures. Patients with KC and sub-clinical KC were recruited from the Affiliated Eye Hospital of Wenzhou Medical University. Complete ocular examinations were performed by an experienced doctor (JJ), including a review of medical and family history, corrected distance visual acuity, slit-lamp biomicroscopy, fundus examination, and corneal topography using the Medmont E300 (Medmont, Inc., Nunawading Melbourne, Australia). The Max-K, Min-K, Avg-K, and Ast-K were recorded.

One eye of each KC patient was included^31^,^42^. The keratoconic eyes were diagnosed by the following clinical findings: (1) at least one of the following slit-lamp signs: stromal thinning, Vogt’s strias, Fleischer’s ring >2-mm arc, or corneal scarring consistent with KC; (2) central average keratometry >47.0 D, asymmetric topographical features with inferior-superior (I-S) value ≥ 2.0 D of the vertical power gradient across the 6-mm region; and (3) no history of contact lens wear, ocular surgery, or extensive scarring. The asymptomatic fellow eye of each patient with unilateral KC was included in the sub-clinical KC group if it had the following features: (1) no clinical signs of KC at slit-lamp biomicroscopy, retinoscopy, and ophthalmoscopy; (2) corneal topographical features with I-S values < 1.4 D of the vertical power gradient across the 6-mm region; and (3) no history of contact lens wear, ocular surgery, or trauma^26^,^38^,^43^. Normal subjects were enrolled among the hospital staff and university students if they met the following screening criteria: (1) corneal topographical features with I-S values < 1.4 D of the vertical power gradient across the 6-mm region; (2) myopia < −6.00 D and astigmatism <−2.00 D; (3) no clinical signs or suggestive topographic patterns for suspicious sub-clinical KC, KC, or pellucid marginal degeneration; (4) no history of ocular surgery or trauma; and (5) stopped contact lens wear for ≥3 weeks for rigid gas permeable and ≥1 week for soft contact lenses.

All of the normal subjects were divided into two groups, Normal Group I and Normal Group II. Normal Group I (51 eyes) was used to set up the standard references of epithelial and Bowman’s layer thicknesses. Normal Group II (30 eyes) was assigned for ROC curve analyses with sub-clinical and manifest KC groups.

### Image Acquisition and Processing

All subjects were imaged using a custom-built UHR-OCT system, which was described previously^25^,^44^,^45^,^46^, and similar to the one used by Abou Shousha *et al.*^14^. Briefly, a superluminescent diode light source with a broad bandwidth of 100 nm centered at a wavelength of 840 nm was used to achieve approximately ~3 μm of axial resolution in corneal tissue. Image acquisition speed was 24 k A-lines per second. Each B-scan consisted of 1,365 × 2,048 pixels, corresponding to a scan depth of 2.02 mm and a scan width of 8.66 mm in the air.

**Figure 3:**
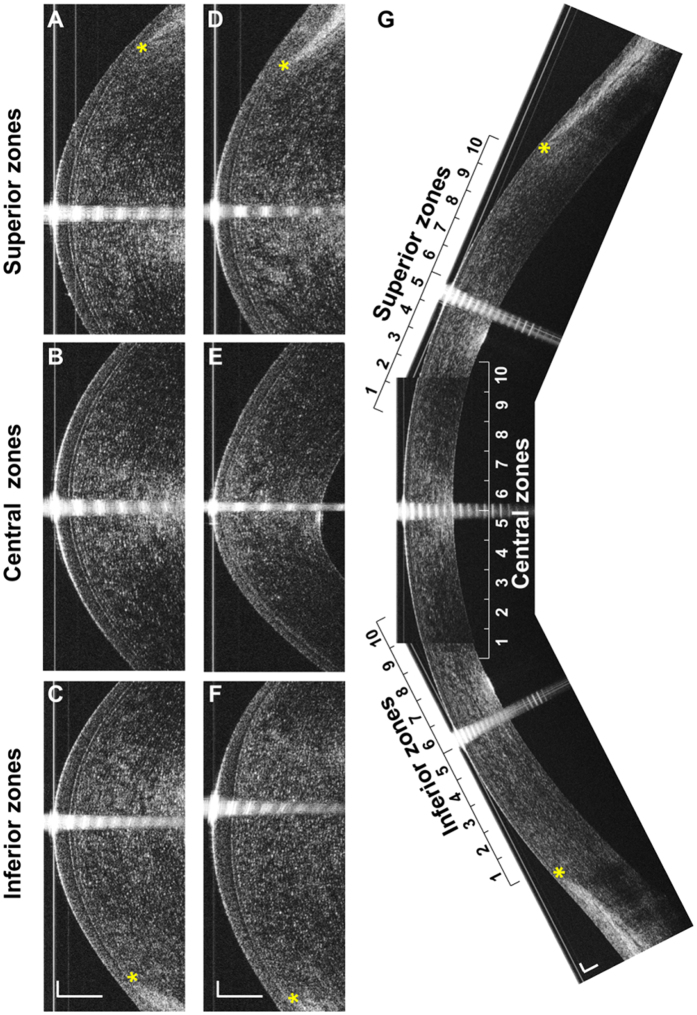
Reconstruction of the entire cornea profile. (**A**) Original superior corneal UHR-OCT image of a normal subject. (**B**) Original central corneal UHR-OCT image of a normal subject. (**C**) Original inferior corneal UHR-OCT image of a normal subject. (**D**) Original superior corneal UHR-OCT image of a KC patient. (**E**) Original central corneal UHR-OCT image of a KC patient. (**F**) Original inferior corneal UHR-OCT image of a KC patient. (**G**) Entire profile reconstruction of a normal cornea. For data analysis, each region was divided into 10 equal zones. The superior and inferior zones ended at the edges of Bowman’s layer. The central zones were centered on the corneal vertex. *the edge point of Bowman’s layer. Bars = 250 μm.

The measurements were performed by an experienced operator (MP) between 10 AM and 4 PM. During OCT imaging, an external visual target was positioned in front of the fellow eye for alignment. A specular reflection of the corneal apex ensured that the OCT scanning probe was aligned perpendicular to the cornea (Fig. 3A–F). Subjects were asked to look straight ahead to center the vertical cornea image (Fig. 3B,E). Each central image was centered on the corneal apex. To image the superior and inferior regions of the cornea, fixation targets were set 15 cm from the subjects with a 30° upward (Fig. 3C,F) and downward angles (Fig. 3A,D).

To obtain the entire vertical thickness profile of the epithelium and Bowman’s layer (Fig. 3G), UHR-OCT image analysis was carried out at Wenzhou Medical University with custom software developed using Matlab (MathWorks, Inc., Natick, MA, USA). The steps of the image analysis were similar to our published papers in which the boundaries of the epithelium and Bowman’s layer were manually outlined^45^,^46^. In the current study, we improved the image processing method by utilizing the gradient information and a shortest path search method^47^,^48^. Thus the boundaries were automatically identified and the layers segmented, as described in our recently published paper^49^. The boundary between Bowman’s layer and the stroma was not as clear as the epithelial layer boundaries, so the procedure for this boundary segmentation started with manual selection of 5–6 different points on the interface. These data points were used to generate an initial estimated boundary using the spline interpolation.

All boundaries detected were then overlaid on the OCT images and visually checked by the grader. If the segmentation misidentified the Bowman’s layer-stromal interface, a manual approach was implemented in the algorithm to correct any minor segmentation errors. Ray tracing based on Snell’s principle was applied to optically correct the position of each boundary because the OCT light was distorted as it passed through the eye^50^,^51^,^52^,^53^. The thickness profiles of the corneal epithelium, Bowman’s layer, and stroma were measured as the distance between the neighboring interfaces perpendicular to the anterior surface. A refractive index of 1.389 was used in calculation^54^.

For central images, the central 1,000 A-scans along the vertical direction, equaled to a 4.23-mm chord distance, were used for data analysis. For peripheral images, 1,000 A-scans from the edge of Bowman’s layer towards the center of the cornea were selected for data analysis. The selected 1,000 A-scans on the central and peripheral images were divided into 10 zones of 100 A-scans each (0.42-mm chord distance). On each image, zones 1 to 10 represented the direction from inferior to superior. The edge of Bowman’s layer on either side served as the standard for co-registration. The reconstructed thickness profiles of the corneal epithelium, Bowman’s layer, and stroma encompassed approximately 11 mm along the vertical meridian.

OCT images of 10 normal subjects and 10 KC patients were randomly chosen to test the repeatability of the epithelium and Bowman’s layer segmentations. The repeatability was defined as the standard deviation of the difference between two measurements by two graders (ZX and MP). For the 10 normal subjects, the repeated measurements between the two graders showed no statistically significant difference. The mean ± standard deviation of the measurement differences of the epithelium thickness was 1.33 ± 0.35 μm and 1.65 ± 0.21 μm over the central and peripheral zones respectively. For Bowman’s layer, the thickness repeatability was 1.28 ± 0.16 μm and 1.23 ± 0.13 μm over the central and peripheral zones respectively. For the 10 KC patients, the repeatability of the epithelium thickness was 1.21 ± 0.50 μm and 1.56 ± 0.35 μm over the central and peripheral zones. For Bowman’s layer measurements, the thickness repeatability was 1.20 ± 0.22 μm and 1.33 ± 0.15 μm over the central and peripheral zones, respectively. The repeatability results of the improved algorithm segmentation were similar to those in our previous reports^45^,^49^.

### Diagnostic Indices Constructed from Vertical Epithelial, Bowman’s Layer, and Stromal Thickness Measurements

To test the diagnostic values of the vertical epithelial, Bowman’s layer, and stromal thickness profiles, indices were built as described in previous studies^14^,^16^ to quantify the different change patterns of these three microstructural layers. Thickness indices of localized thinning for the corneal epithelium, Bowman’s layer, and stroma were calculated as the EEI, BEI, and SEI respectively. Maximun ectasia indices, EEI-MAX, BEI-MAX, and SEI-MAX, were also calculated for the same three layers. Root-mean-square variations of the zonal thicknesses and profile averages within each subject were calculated as EPV, BPV, and SPV, respectively. Root-mean-square deviations from the zonal thicknesses of individual profiles and pattern average were calculated as EPSD, BPSD, and SPSD, respectively, which showed the difference between an individual thickness profile and the pattern profile of the average thickness from normal subjects. Detailed definition and the significance of each index were described in Table 3.

### Statistical Analysis

All data analyses were performed by the Statistical Package for the Social Sciences software (ver. 17, SPSS, Inc., Chicago, IL, USA). The means, standard deviations, and 95% confidence intervals (CI) were calculated for all continuous variables. The Kolmogorov-Smirnov test was used to determine the normality of the distribution for each variable. Comparisons among normal, sub-clinical KC, and KC groups were made using ANOVA. P < 0.05 was defined as the level of statistical significance.

To find the lowest possible number of independent matrices for correct discrimination, linear stepwise discriminant analysis was applied to build discriminant functions with individual indices obtained from the epithelium, Bowman’s layer, and stroma. Indices with the smallest Wilk’s λ and an F > 3.84 returned from an intergroup ANOVA were included in the function. The predictive accuracy of each individual index and the output values of the discriminant function in differentiating between patients with KC and normal eyes and between patients with sub-clinical KC and normal eyes was determined by ROC curves. An AUC of 100% implied perfect diagnostic performance^55^.

## Additional Information

**How to cite this article**: Xu, Z. *et al.* Value of corneal epithelial and Bowman’s layer vertical thickness profiles generated by UHR-OCT for sub-clinical keratoconus diagnosis. *Sci. Rep.* **6**, 31550; doi: 10.1038/srep31550 (2016).

## Figures and Tables

**Table 1:** Clinical information for normal, sub-clinical keratoconus, and keratoconus groups.

	Normal (n = 81)	Sub-KC (n = 32)	KC (n = 37)
SE (D)	−3.78 ± 2.23	−3.75 ± 2.78	−7.72 ± 4.24*
BCVA (decimal VA)	1.1 ± 0.1	1.0 ± 0.1	0.5 ± 0.3*
Max-K (D)	44.2 ± 1.5	44.1 ± 1.0	54.0 ± 7.9*
	(95% CI: 43.9–44.5)	(95% CI: 43.7–44.5)	(95% CI: 51.5–56.5)
Min-K (D)	43.0 ± 1.5	42.9 ± 1.0	48.6 ± 6.7*
	(95% CI: 42.7–43.3)	(95% CI: 42.5–43.3)	(95% CI: 46.4–50.8)
Avg-K (D)	43.6 ± 1.4	43.5 ± 1.0	51.3 ± 7.2*
	(95% CI: 43.3–43.9)	(95% CI: 43.1–43.9)	(95% CI: 49.0–53.6)
Ast-K (D)	1.2 ± 0.7	1.2 ± 0.7	5.4 ± 3.5*
	(95% CI: 1.0–1.4)	(95% CI: 0.9–1.5)	(95% CI: 4.3–6.5)

Normal, normal group; Sub-KC, sub-clinical keratoconus group; KC, keratoconus group; n, number of eyes; SE, spherical equivalent; BCVA, best corrected visual acuity; Max-K, maximum keratometry; Min-K, minimum keratometry; Avg-K, average keratometry; Ast-K, astigmatic keratometry; 95% CI, 95% confidence interval; VA, visual acuity; D, diopter; *P < 0.05 compared to the normal group.

**Table 2:** Regional thicknesses of the corneal epithelium, Bowman’s layer, and stroma.

	Normal (μm)	Sub-KC (μm)	KC (μm)
Epithelium			
Superior region	50.91 ± 3.09	50.27 ± 3.02	51.09 ± 4.29
Central region	53.48 ± 2.83	51.92 ± 2.57*	46.10 ± 5.31*
Inferior region	54.94 ± 2.80	54.85 ± 3.36	54.45 ± 5.03
Bowman’s layer			
Superior region	17.18 ± 1.96	16.58 ± 1.75	16.23 ± 2.80
Central region	17.07 ± 1.51	16.02 ± 2.10*	12.06 ± 2.50*
Inferior region	17.08 ± 1.35	15.70 ± 1.62*	15.23 ± 2.12*
Stroma			
Superior region	525.27 ± 32.64	512.04 ± 40.82	493.15 ± 32.85*
Central region	460.32 ± 29.65	454.16 ± 36.25	412.49 ± 39.19*
Inferior region	533.30 ± 29.03	509.67 ± 38.32*	482.35 ± 38.54*

Normal, normal group; Sub-KC, sub-clinical keratoconus group; KC, keratoconus group; Superior region, 4.23 mm from the superior edge of Bowman’s layer; Central region, central 4.23-mm diameter through the corneal apex; Inferior region, 4.23 mm from the inferior edge of Bowman’s layer; *P < 0.05 compared to the normal group.

**Table 3:** Definitions and significance of indices based on vertical thickness profiles of the corneal epithelium, Bowman’s layer, and stroma.

Indices	Definitions	Significance
EEI, BEI, SEI	Minimum thickness in the inferior half divided by the average thickness in the superior half multiplied by 100	Localized thinning in the vertical meridian
EEI-MAX, BEI-MAX, SEI-MAX	Minimum thickness in the inferior half divided by the maximum thickness in the superior half multiplied by 100	Maximum localized thinning in the vertical meridian
EPV, BPV, SPV	Root mean square between zonal thicknesses and profile average within one subject	Variation of thickness profile within each individual
EPSD, BPSD, SPSD	Root mean square of the zonal thicknesses of individual profiles and zonal thicknesses of pattern average	Standard deviation of thickness profile between individual and normal pattern

EEI, epithelium ectasia index; BEI, Bowman’s layer ectasia index; SEI, stroma ectasia index; EEI-MAX, maximum epithelium ectasia index; BEI-MAX, maximum Bowman’s layer ectasia index; SEI-MAX, maximum stroma ectasia index; EPV, epithelium profile variation; BPV, Bowman’s layer profile variation; SPV, stroma profile variation; EPSD, epithelium profile standard deviation; BPSD, Bowman’s layer profile standard deviation; SPSD, stroma profile standard deviation.

Zonal thickness, the averaged thickness of 100 A-scans (0.42-mm chord distance) of each zone on the UHR-OCT B-scan image.

**Table 4:** Diagnostic indices of epithelium, Bowman’s layer and stroma for sub-clinical keratoconus and keratoconus.

Indices	Normal	Sub-clinical KC						KC					
	mean	mean	P	AUC	cut off	Sen (%)	Spe (%)	mean	P	AUC	cut off	Sen (%)	Spe (%)
Epithelium													
EEI (%)	100.36 ± 3.11	92.06 ± 5.74	<0.001	0.928	97.82	91	87	76.11 ± 10.75	<0.001	1.000	93.88	100	100
EEI-MAX (%)	94.22 ± 2.76	85.01 ± 6.31	<0.001	0.939	91.13	88	90	65.92 ± 12.00	<0.001	1.000	83.52	100	100
EPV (μm)	2.87 ± 0.63	3.94 ± 1.04	<0.001	0.798	3.21	81	77	7.43 ± 2.64	<0.001	0.983	4.12	96	97
EPSD (μm)	3.03 ± 1.18	4.17 ± 1.35	<0.001	0.754	3.54	66	70	8.56 ± 2.92	<0.001	0.987	4.29	100	97
Bowman’s layer													
BEI (%)	89.18 ± 6.62	77.10 ± 8.61	<0.001	0.870	84.10	81	77	59.50 ± 14.42	<0.001	0.980	75.49	89	100
BEI-MAX (%)	78.55 ± 5.80	65.92 ± 8.89	<0.001	0.892	74.22	84	77	49.18 ± 13.48	<0.001	0.977	65.03	89	100
BPV (μm)	1.30 ± 0.28	1.83 ± 0.45	<0.001	0.847	1.62	69	87	2.67 ± 0.99	<0.001	0.945	1.71	84	97
BPSD (μm)	1.58 ± 0.51	2.48 ± 0.81	<0.001	0.766	2.02	72	73	3.88 ± 1.38	<0.001	0.934	2.85	94	97
Stroma													
SEI (%)	88.73 ± 2.15	88.93 ± 1.63	>0.05	0.529	89.27	70	53	80.33 ± 6.06	<0.001	0.944	86.91	95	83
SEI-MAX (%)	73.02 ± 3.62	73.80 ± 2.39	>0.05	0.430	73.84	49	47	64.91 ± 6.73	<0.001	0.873	69.90	78	80
SPV (μm)	53.97 ± 8.42	48.39 ± 5.62	>0.05	0.306	50.65	42	40	59.35 ± 10.90	=0.01	0.678	55.88	65	70
SPSD (μm)	28.05 ± 16.03	35.48 ± 21.30	>0.05	0.586	31.73	55	70	54.58 ± 25.05	<0.001	0.832	38.11	76	83

Normal, normal group; Sub-KC, sub-clinical keratoconus group; KC, keratoconus group; AUC, area under receiver operating characteristic curve; EEI, epithelium ectasia index; EEI-MAX, maximum epithelium ectasia index; EPV, epithelium profile variation; EPSD, epithelium profile standard deviation; BEI, Bowman’s layer ectasia index; BEI-MAX, maximum Bowman’s layer ectasia index; BPV, Bowman’s layer profile variation; BPSD, Bowman’s layer profile standard deviation; SEI, stroma ectasia index; SEI-MAX, maximum stroma ectasia index; SPV, stroma profile variation; SPSD, stroma profile standard deviation; Sen, sensitivity; Spe, specificity; P, P-value.
